# Genome Editing for Crop Improvement – Applications in Clonally Propagated Polyploids With a Focus on Potato (*Solanum tuberosum* L.)

**DOI:** 10.3389/fpls.2018.01607

**Published:** 2018-11-13

**Authors:** Satya Swathi Nadakuduti, C. Robin Buell, Daniel F. Voytas, Colby G. Starker, David S. Douches

**Affiliations:** ^1^Department of Plant, Soil and Microbial Sciences, Michigan State University, East Lansing, MI, United States; ^2^Department of Plant Biology, Michigan State University, East Lansing, MI, United States; ^3^Plant Resilience Institute, Michigan State University, East Lansing, MI, United States; ^4^AgBioResearch – Michigan State University, East Lansing, MI, United States; ^5^Department of Genetics, Cell Biology, and Development, Center for Precision Plant Genomics, University of Minnesota, Saint Paul, MN, United States

**Keywords:** genome-editing, clonal propagation, polyploidy, potato (*Solanum tuberosum*), CRISPR/Cas system, TALENs, *Agrobacterium*-mediated transformation, protoplast transformation

## Abstract

Genome-editing has revolutionized biology. When coupled with a recently streamlined regulatory process by the U.S. Department of Agriculture and the potential to generate transgene-free varieties, genome-editing provides a new avenue for crop improvement. For heterozygous, polyploid and vegetatively propagated crops such as cultivated potato, *Solanum tuberosum* Group Tuberosum L., genome-editing presents tremendous opportunities for trait improvement. In potato, traits such as improved resistance to cold-induced sweetening, processing efficiency, herbicide tolerance, modified starch quality and self-incompatibility have been targeted utilizing CRISPR/Cas9 and TALEN reagents in diploid and tetraploid clones. However, limited progress has been made in other such crops including sweetpotato, strawberry, grapes, citrus, banana etc., In this review we summarize the developments in genome-editing platforms, delivery mechanisms applicable to plants and then discuss the recent developments in regulation of genome-edited crops in the United States and The European Union. Next, we provide insight into the challenges of genome-editing in clonally propagated polyploid crops, their current status for trait improvement with future prospects focused on potato, a global food security crop.

## Introduction

Genome-editing technologies such as TALENs (Transcription Activator Like Effector Nucleases), CRISPR/Cas9 (Clustered Regularly Interspaced Short Palindromic Repeats/CRISPR-associated systems), CRISPR/Cas12a (Cpf1, CRISPR from *Prevotella* and *Francisella* 1), and Cas9-derived DNA base editors, provide an unprecedented advancement in genome engineering due to precise DNA manipulation. Genome-editing is being widely applied in plants and has revolutionized crop improvement. Polyploidy and vegetative reproduction are unique to plants, frequently found in a large number of important food crops including root and tuber crops, several perennial fruit crops as well as forage crops (McKey et al., 2010; Gemenet and Khan, 2017). Several cultivated polyploids have vegetative mode of reproduction (Herben et al., 2017) and with allopolyploidy combined with heterozygosity makes breeding challenging in these crops. In order to introduce genetic diversity by crossing two heterozygous parents, multiple alleles segregate at a given locus. Backcrossing techniques to add traits cannot be used because it will destroy the unique gene combination within a preferred variety.

Potato, (*Solanum tuberosum* Group Tuberosum L.) (2*n* = 4*x* = 48) represents one such heterozygous, polyploid crop that is clonally propagated by tubers. Potato is a global food security crop and is the third most important food crop after rice and wheat (Devaux et al., 2014). While conventional breeding and genetic analysis are challenging in cultivated potato due to the above mentioned features, majority of diploid potatoes possess gametophytic self-incompatibility (SI). Historically, conventional breeding has been used to create improved potato cultivars. Yet due to its unique challenges, breeding is inefficient when a large number of agronomic, market quality and resistance traits need to be combined or if novel traits not present in the germplasm bank are wanted. Insertion and expression or silencing of economically important genes is being used to improve potato production and quality traits without impacting optimal allele combinations in current varieties (Diretto et al., 2006, 2007; Rommens et al., 2006; Chi et al., 2014; Clasen et al., 2016; Sun et al., 2016; Andersson et al., 2017; McCue et al., 2018). Genome sequence information coupled with established genetic transformation and regeneration procedures make potato a strong candidate for genetic engineering. In 2017, the U.S. Department of Agriculture’s (USDA) Animal and Plant Health Inspection Service (APHIS), the Environmental Protection Agency (EPA) and the Food and Drug Administration (FDA) approved Simplot Plant Sciences to commercially release genetically engineered potatoes with reduced bruising and acrylamide content in tubers (Innate potatoes^1^).

In this review, we describe various genome-editing platforms available for plants, their delivery mechanisms and discuss the recent USDA and the European Union clarifications regarding regulatory aspects of gene-edited crops. Next, we discuss the challenges of genome-editing in clonally propagated polyploid crops and summarize the insights gained from case studies along with future prospects focused on enhancement of potato breeding using this technology.

## Genome-Editing – Emerging Technologies for Genetic Manipulation in Plants

**FIGURE 1:**
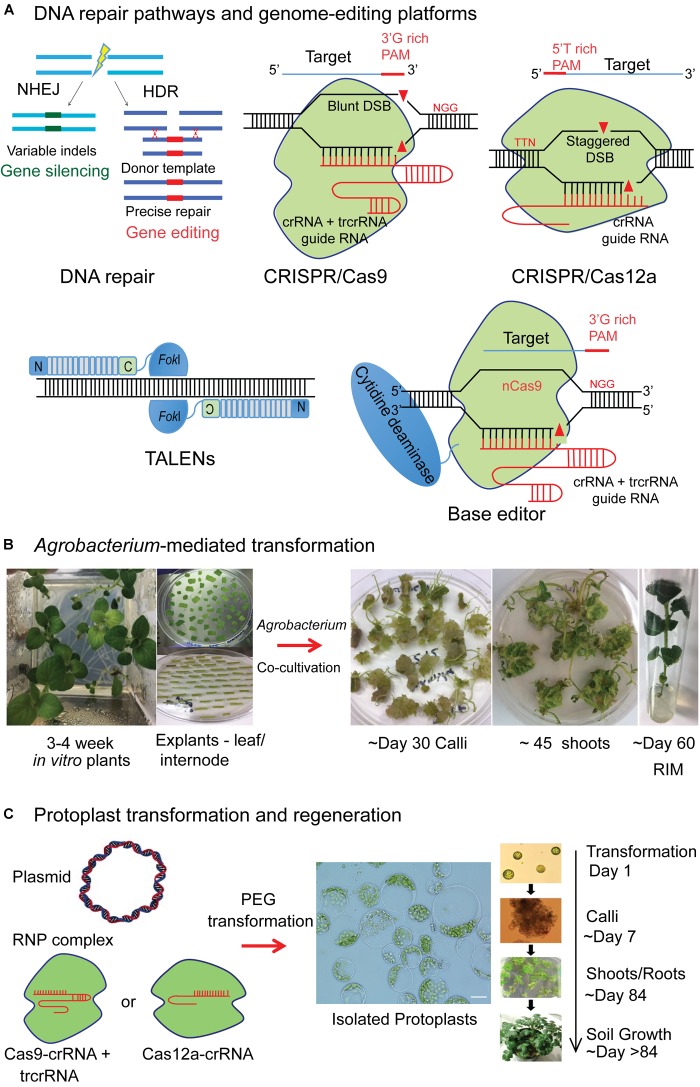
Illustration of genome-editing platforms and genetic transformation procedures in potato. **(A)** Double stranded DNA (dsDNA) break repair in a cell occurs either by non-homologous end joining (NHEJ), where the cleaved DNA molecule is simply rejoined, often with indels in coding regions (green) that result in gene knock-out or by homologous recombination (HR), where a donor repair template (red) can be used for targeted knock-in experiments, where a single or few nucleotides alterations, insertion of an entire transgene or suites of transgenes can be made. CRISPR/Cas9 nuclease engineered to have a Cas9 protein and a guide RNA (gRNA) that is a fusion of CRISPR RNA (crRNA) and *trans*-activating crRNA (tracrRNA). Cas9 and gRNA complex can recognize and cleave target dsDNA that is complementary to 5′ end of target spacer sequence that is next to protospacer adjacent motif (PAM) of 5′-NGG-3′. CRISPR/Cas12a is a single CRISPR RNA guided nuclease lacking tracrRNA. Cas12a has PAM requirement of “TTTN” allowing targeting of AT rich regions and expanding the target range of RNA-guided genome-editing nucleases. Cas12a cleaves DNA at sites distal to PAM and introduces a staggered DSB with a 4–5-nt 5′ overhang, unlike blunt DSB by Cas9. Transcription activator-like effector nucleases (TALENs) bound to their target site are shown. The TALE array contains repeat variable di-residues that make sequence-specific contact with the target DNA. TALE repeats are fused to *Fok*I, a non-specific nuclease that can cleave the dsDNA upon dimerization. Base editor constitutes fusion of nickase Cas9 (nCas9) with cytidine deaminase enabling the editing of single bases by C→T conversion of single-stranded target. **(B)** *Agrobacterium*-mediated plant transformation and regeneration in potato. 3–4-week-old *in vitro* propagated potato plants in a Magenta box are shown. Ex-plants are prepared from leaf and stem internodes and placed on callus induction media after *Agrobacterium* inoculation and co-cultivation. Callus growth observed from the ex-plants. After 6–8 weeks, shoots emerge and are grown on shoot induction media. 1–2 cm shoots are excised and transferred to root induction media. The lines that develop roots and have growth on selection media are chosen as candidates for molecular screening to confirm the gene editing events. **(C)** Delivery of the gene editing reagents as plasmid DNA or as preassembled Cas9 or Cas12a protein-gRNA ribonucleoproteins (RNPs) into protoplasts by polyethylene glycol (PEG) mediated transformation. The timeline from protoplast transformation to regeneration of mutagenized plants in potato is reproduced from Clasen et al. (2016) with the permission of the copyright holder (John Wiley & Sons, Inc.).

Genome-editing by sequence-specific nucleases (SSNs) such as CRISPR/Cas9 and TALENs facilitate targeted insertion, replacement, or disruption of genes in plants. SSNs create double stranded breaks (DSBs) at the target locus and rely on cellular repair mechanisms to correct these breaks (Figure 1A).

The CRISPR/Cas9 system has demonstrated great potential in various crop species due to simplicity of use and versatility of the reagents (Jiang W. et al., 2013; Sun et al., 2015; Svitashev et al., 2016; Zhang et al., 2016; Shimatani et al., 2017; Soyk et al., 2017). Engineered CRISPR/Cas9 nucleases target DNA adjacent to the 5′-NGG-3′, protospacer adjacent motif (PAM), in a single guide RNA (sgRNA) specific manner (Jinek et al., 2012; Figure 1A). CRISPR/Cas12a that has PAM requirement of “TTTN”, allowing targeting of AT rich regions, is emerging as equally effective alternative, implemented in various plants (Kim et al., 2017; Tang et al., 2017). For multiplexing, to target more than one gene at a time, Cas12a requires only a single RNA PolIII promoter to drive several crRNAs, whereas Cas9 requires relatively large constructs (Zetsche et al., 2017). In TALENs, the TALE protein is engineered for sequence-specific DNA binding and is fused to a non-sequence-specific *Fok*I nuclease to create a targeted DSB (Bogdanove and Voytas, 2011; Voytas, 2013).

Base-editing technology, based on CRISPR/Cas9 system generates base substitutions without requiring dsDNA cleavage. Cas9 is engineered to retain DNA-binding ability in a sgRNA programmed manner without the nuclease activity such as catalytically inactive Cas9 (dCas9) or a nickase (nCas9) (Jinek et al., 2012). If either dCas9 or nCas9 is fused with a cytidine deaminase that mediates the conversion of cytidine to uridine, the result is a base editor that results in a C→T (or G→A) substitution (Komor et al., 2016; Figure 1A). More recently, adenine base editors have been developed that convert A→G (or T→C) (Gaudelli et al., 2017). Base editing has been successfully applied in plants to confer both gain of function by incorporating correct mutations and loss of function by generating knock-out mutations (Chen et al., 2017; Li et al., 2017; Lu and Zhu, 2017; Shimatani et al., 2017; Zong et al., 2017; Kang et al., 2018).

## Delivery of Genome-Editing Nucleases Into Plant Cells

The three major methods of genetic transformation in plants are: *Agrobacterium-*mediated transformation, biolistics and protoplast transfection. By far the most commonly used method to introduce genome-editing reagents in potato is by *Agrobacterium-*mediated transformation (Figure 1B). A binary T-DNA vector is used to deliver and express the reagents in plant cells. Once inside the nucleus, the T-DNA randomly integrates into the plant/host genome leading to stable transformation resulting in persistent activity of reagents. However, there is a possibility that it remains extra-chromosomal leading to transient gene expression.

The other common method is polyethylene glycol (PEG)-mediated protoplast transfection. Protoplasts facilitate direct delivery of DNA into cells with gene-editing reagents expressed as plasmid DNA for transient transformation. Protoplasts have greater transformation efficiency compared to other methods (Jiang F. et al., 2013; Dlugosz et al., 2016; Baltes et al., 2017). They retain their cell identity and differentiated state and, for some plant species, have the capability to regenerate into an entire plant.

To improve specificity and to reduce the duration of activity of SSNs in the cell, purified recombinant Cas9 or Cas12a protein with an *in vitro* transcribed or synthetically produced sgRNA resulting in a ribonucleoprotein complex (RNP) is delivered into protoplasts (Figure 1C). The Cas9 protein continues to be expressed in the cell for several days when delivered as a plasmid, whereas it is degraded within 24 h when delivered as RNPs, improving the specificity of the reagent (Zetsche et al., 2015). Preassembled CRISPR/Cas9 or Cas12a RNP complexes were successfully delivered into protoplasts of Arabidospsis, tobacco, lettuce, rice, wheat, soybean and potato and plants were regenerated with heritable targeted mutagenesis (Woo et al., 2015; Kim et al., 2017; Liang et al., 2017; Andersson et al., 2018). Using RNPs, possibility of integration of plasmid-derived DNA sequences or foreign DNA into the host genome can be eliminated. Plants regenerated from protoplast cells without the integration of any foreign DNA would likely avoid the regulatory process (Haun et al., 2014; Clasen et al., 2016).

## Regulatory Aspects on Genome-Edited Crops – Impact on Advancing Crop Improvement

Genome-editing has been successfully implemented in several plant species, and some cases, the regulatory status of the edited plants has been considered by USDA/APHIS (“Am I Regulated?”^2^ (Waltz, 2018). USDA considers genome-editing as a novel breeding tool and released a definitive statement that if plant varieties developed through genome-editing do not possess any foreign genetic material and they are indistinguishable from those developed by conventional breeding or mutagenesis approaches, then they will not be regulated (USDA press release^3^). The edits made in edited varieties can include deletions of any length, single base substitutions or genetic variation from any species or variety that is sexually compatible. In the case of *Agrobacterium*-mediated delivery of SSNs, any stably integrated T-DNA sequences can be segregated away by meiotic recombination. Null segregants – progeny of the transgenic, edited parent that still retain the germline edit but lack the integrated T-DNA or other foreign sequence – are excempt from regulation. In clonally propagated plants like potato, null segregants are difficult or impossible to obtain. However, transient expression in protoplasts, for example, can achieve gene edits, and regeneration of the edited protoplasts can create edited plants without any foreign DNA and hence are exempt from regulation by USDA/APHIS (Clasen et al., 2016) (“Am I Regulated?”^2^). In Japan, a government panel recently recommended following a regulatory policy similar to that of USDA/APHIS, that gene edited plants in Japan should not be regulated (TheScientist news^4^).

Clarity on guidelines for regulating gene-edited crops will undoubtedly promote wider use of this technology in the United States. In contrast, the European Union recently declared that plants generated by genome-editing are not exempt from regulation; rather, they must be treated just like transgenic plant lines (Court of Justice of the European Union verdict^5^). The EU’s argument is that gene editing alters the genetic material in a way that is not natural, and edited plants might have adverse effects on human health and the environment. Unlike the United States, Europe chose a “process-based” approach to regulation, rather than a “product-based” approach. Gene editing could be used to create genetic variation that is identical to that already present in crop varieties grown in Europe; however, it would nonetheless be regulated due to this process-based approach. A “product-based” regulatory policy allows multiple levels of checks and balances. For example, in the United States, the FDA can weigh in on health benefits or concerns of a given crop, and the EPA can weigh in on potential environmental effects of an edited plant variety. The conservative, process-based approach adopted by Europe will likely both slow the development of the technology in European research labs and will also have global ramifications in terms of trade of gene edited commodities.

## Genome-Editing Challenges in Clonally Propagated Polyploid Crops – Case Studies in Potato

Genome manipulation in polyploid heterozygous crops include the task of simultaneously targeting multiple alleles and screening large number of transformants to recover multiallelic mutagenic lines. Moreover, unlike the seed producing species where Cas9 can be segregated out, it is not feasible in clonally propagated plants. Nevertheless, genome-editing using TALENs and CRISPR/Cas9 has been successfully demonstrated in a number of clonally propagated crops presented in Table 1. Potato is chosen for case studies, since it has been subjected to more genome-editing, even though it is a tetraploid, compared to other crops.

**Table 1:** Genome editing case studies in clonally propagated crops.

Potato	Ploidy	Genome editing	Translormation method	Target gene	Trait associated with the gene	Purpose of the study	Reference
*S.tuberosum* cv. Sassy	4x	TALENs	Agrobacterium	*Sterol side chain reductase 2 (StSSR2)*	Steroidal glycoalkaloids reduction in tuber	Identify key enzyme in the biosynthesis of cholesterol and related steroidal glycoalkaloids	Sawai et al., 2014
*S.tuberosum* cv. Desiree	4x	TALENs	Protoplasts	*Acetolactate synthase1 (StALS1)*	Herbicide resistance	Transient expression of TALENs in potato protoplasts for targeted mutagenesis and regeneration	Nicolia et al., 2015
*S.tuberosum* cv. Ranger Russet	4x	TALENs	Protoplasts	*Vacuolar invertase (StVlnv)*	cold induced sweetening, acrylamide content in tubers	Tuber improvement for cold storage	Clasen et al., 2016
*S.tuberosum* cv. Ranger Russet	4x	TALENs	Agrobacterium	*StALS1*	Herbicide resistance	Use of TALENs for targeted T-DNA integration	Forsyth et al., 2016
*S.tuberosum* cvs. Russet Burbank, Shepody	4x	TALENs	Agroinfiltration	*1,4-alpha-glucan branching enzyme gene (SBE1), StVInv*	Degree of starch branching, cold induced sweetening	Rapid testing and effective delivery of TALENs	Ma et al., 2017
*S.tuberosum* cv. Kuras	4x	CRISPR/Cas9	Protoplasts	*Granule-bound starch synthase (StGBSS)*	Tuber starch quality	Transient expression of CRISPR/Cas9 in potato protoplasts for targeted mutagenesis and regeneration. Potato tuber with altered starch content developed	Andersson et al., 2017
*S.tuberosum* cv. Kuras	4x	CRISPR/Cas9 RNPs	Protoplasts	*StGBSS*	Tuber starch quality	Use of RNPs for genome-editing in potato protoplasts and regeneration of mutant lines with knock-out of all four alleles	Andersson et al., 2018
*S.tuberosum* cv. Desiree	4x	CRISPR/Cas9	Agrobacterium	*Transcription factor gene StMYB44*	Phosphate transport via roots	Understand the molecular basis of phosphate stress responses in potato	Zhou et al., 2017
MSX914-10, *S. tuberosum*	2x, 4x	CRISPR/Cas9	Agrobacterium GVR	*StALS1*	Herbicide resistance	Targeted mutagenesis in potato and germline inheritance	Butler et al., 2015
MSX914-10, *S. tuberosum* cv. Desiree	2x, 4x	CRISPR/Cas9, TALENs	Agrobacterium GVR	*StALS1*	Herbicide resistance	Gene targeting via homologous recombination using donor template and geminivirus replicons	Butler et al., 2016
*S. tuberosum* Group Phureja double monoploid	2x	CRISPR/Cas9	Agrobacterium	*StlAA2*	Petiole hyponasty and shoot morphogenesis	Targeted mutagenesis using native StU6 promoter driving the sgRNA	Wang et al., 2015
*S. tuberosum* Group Phureja S15-65	2x	CRISPR/Cas9	Agrobacterium	*Stylar ribonuclease gene (S-Rnase)*	Self Incompatibility	Knock-out of self-incompatibility gene *S-RNase* in diploid potato line resulted in self compatibility	Ye et al., 2018
No mention	N/A	TALENs	Agrobacterium	*StGBSS*	Tuber starch quality	Development of a Gateway system for rapid assembly of TALENs in a binary vector	Kusano et al., 2016
**Cassava**							
*Manihot esculenta* cvs. 60444 and TME204	2x	CRISPR/Cas9	Agrobacterium	*Phytoene desaturase* (*MePDS*)	Albino devoid of green tissue	Targeted mutagensis in Cassava	Odipio et al., 2017
*Manihot esculenta* cv. 60444	2x	CRISPR/Cas9	Agrobacterium	*novel cap-binding proteins (nCBP-1.nCBP-2)*	Cassava brown streak disease (CBSD)	Delayed viral disease incidence and reduced severity of storage root necrosis	Gomez et al., 2018
**Apple**							
*Malus domestica* cv. Golden delicious	2x	CRISPR/Cas9 RNPs	Protoplasts	*DspE-interacting proteins of Malus(DIPM 1-4)*	Fire blight resistance	Use of RNPs for genome-editing in apple protoplasts	Malnoy et al., 2016
*Malus domestica* cv. JM2	2x	CRISPR/Cas9	Agrobacterium	*MdPDS*	Albino devoid of green tissue	Targeted mutagensis in apple	Nishitani et al., 2016
**Grape**							
*Vitis vinifera* cv. Chardonnay	2x	CRISPR/Cas9 RNPs	Protoplasts	*Mildew Locus* 0 *(VvMLO-7)*	Powdery mildew resistance	Use of RNPs for genome-editing in grape protoplasts	Malnoy et al., 2016
*Vitis vinifera* cv. Chardonnay	2x	CRISPR/Cas9	Agrobacterium	L-*idonate dehydrogenase gene (IdnDH)*	Biosynthesis of tartaric acid	Use of grape suspension cells for genome-editing resulting in reduced tartaric acid production	Ren et al., 2016
*Viti*s *vinifera* cv. Thompson Seedless	2x	CRISPR/Cas9	Agrobacterium	*VvWRKY52*	*Botrytis cinerea* resistance	Use of grape suspension cells for genome-editing resulting in Botrytis resistance	Wang et al., 2018
*Vitis vinifera* cv. Neo Muscat	2x	CRISPR/Cas9	Agrobacterium	*VvPDS*	Albino devoid of green tissue	Genome-editing using grape embryonic callus	Nakajima et al., 2017
**Banana**							
*Musa spp* cv. Williams (AAA Cavendish), Rasthali (AAB Silk) (AAB Silk)	3x	CRISPR/Cas9	Agrobacterium	*MaPDS*	Albino devoid of green tissue	Genome-editing using banana embryonic callus achieving tri-allelic mutations	Kaur et al., 2018; Naim et al., 2018
**Sweet orange**							
*Citrus sinensis* Osbeck *Citrus paradisi* (Duncan grape fruit)	2x	CRISPR/Cas9	Agrobacterium	*Lateral organ boundaries 1 (CsLOB1)*	Citru canker (*Xanthomonas citri)* resistance	Demonstrated that editing promoter or coding region of *CsLOB1* resulted in canker-resistant citrus cultivars	Peng et al., 2017; Jia et al., 2016, 2017
*Citrus sinensis* cv. Valencia Carrizo Citrange *[Poncirus trifoliata L. Raf.*x *Citrus sinensis* L Osb)	2x	CRISPR/Cas9	Agroinfiltration Agrobacterium	*CsPDS*	Albino devoid of green tissue	Demonstrated that *Xanthomonas* citri pretreatent before Agroinfiltration targeting *PDS* resulted in albino phenotype and Cas9 driven by *A.thaliana* YAO promoter has enhanced targeteged mutagenesis compared to CaMV35S promoter	Jia and Nian, 2014; Zhou et al., 2018
**Sugarcane**							
*Saccharum spp* cv. CP88-1762	10-13x	TALENs	Agrobacterium Biolistics	*caffeic acid O-methyltransferase (SoCOMT)*	Reduction in lignin content	Produce lignocellulosic ethanol from sugacane by altering the cell wall properties	Jung and Altpeter, 2016
**Strawberry**							
*Fragaria vesca*	2x	CRISPR/Cas9	Agrobacterium	*Tryptophan aminotransferase 1* (*TAA1*), *Auxin response factor (FveARFB)*	Auxin biosynthesis and response	Targeted mutagenesis and germ-line inheritance in strawberry as a proof of concept	Zhou et al., 2018
^∗^RNP = Ribonucleo protein	GVR = geminivirus replicon						

The first successful demonstration of the use of TALENs in a tetraploid potato cultivar was by knocking out all four alleles of *Sterol side chain reductase 2* (*StSSR2*) (Sawai et al., 2014) involved in anti-nutritional sterol glycoalkaloid (SGA) synthesis (Itkin et al., 2011, 2013). Similarly, using CRISPR/Cas9 and TALENs, geminivirus replicon-mediated gene targeting (by HR) was successfully demonstrated in diploid and tetraploid varieties. The endogenous *Acetolactate synthase1* (*StALS1*) gene was modified to incorporate mutations using a donor repair template leading to herbicide tolerance and mutations were shown to be heritable (Butler et al., 2015, 2016). *StALS1* was also targeted by TALENs via protoplast transfection and successful regeneration of *StALS1* knock-out lines from transformed protoplasts was demonstrated in tetraploid potato (Nicolia et al., 2015). Initial studies in potato mainly constituted proof-of-concept demonstrations of the genome-editing technology.

However, improvement in tuber cold storage quality of a commercial tetraploid cultivar, Ranger Russet, was achieved by targeting *Vacuolar invertase* (*StVlnv*) using TALENs via protoplast transformation and regeneration (Clasen et al., 2016). Vlnv enzyme breaks down sucrose to the reducing sugars glucose and fructose in cold-stored potato tubers which form dark-pigmented bitter tasting products when processed at high temperatures (Sowokinos, 2001; Kumar et al., 2004; Matsuura-Endo et al., 2006). In addition, the reducing sugars react with free amino acids via the nonenzymatic Maillard reaction to form acrylamide, a carcinogen (Tareke et al., 2002). Tubers from *StVlnv* knock-out lines had undetectable levels of reducing sugars, low acrylamide, and made light colored chips along with no foreign DNA in their genome (Clasen et al., 2016). Recently, a waxy potato with altered tuber starch quality was developed by knocking out all four alleles of *Granule-bound starch synthase* (*GBSS*) in a tetraploid potato cultivar via CRISPR/Cas9. By transient expression of reagents as plasmid DNA or via RNPs in potato protoplasts, mutagenized lines in all four alleles were regenerated with tubers that had the desired high amylopectin starch (Andersson et al., 2017, 2018).

Furthermore, studies related to technological advances in genome-editing in potato have been reported such as utilizing a native StU6 promoter to drive sgRNA expression, targeted insertion of transgenes, a Gateway system for rapid assembly of TALENs, and delivery of TALENs via agroinfiltration for rapid mutagenesis detection (Wang et al., 2015; Forsyth et al., 2016; Kusano et al., 2016; Ma et al., 2017).

## Future Prospects to Enhance Potato Breeding Using Genome-Editing

Genome-editing has tremendous potential for crop improvement, and although implemented in many crops, it has yet to be fully realized in clonally propagated polyploids like potato. Only certain cultivars of potato are amenable to transformation and others need to be tested for transformation and regeneration in tissue culture. Protoplast transformation and regeneration of plants from leaf protoplasts also can lead to somaclonal variation, which may have negative impact(s) on plant development.

In potato, Late blight, caused by fungus *Phytophthora infestans*, is the most critical problem and threat to global potato production (Fry, 2008; Fisher et al., 2012). Two approaches currently used to combat this disease are fungicide spraying and breeding for disease resistance. Canonical disease resistance genes, *R*-genes, belong to nucleotide-binding, leucine-rich repeat (NLR) class of intracellular immune receptor proteins that recognize pathogen effectors to initiate defense responses in the plant (El Kasmi and Nishimura, 2016; Jones et al., 2016). Due to continued high rates of evolution of effector proteins, pathogens overcome recognition, thereby limiting the durability of resistance (Raffaele et al., 2010; Dong et al., 2014). Genome-editing by base editors could potentially be applied to engineer potato for late blight resistance by editing the codons encoding specific amino acids in *R*-genes essential for effector recognition.

Loss of susceptibility is considered as an alternative breeding strategy for durable broad spectrum resistance (Pavan et al., 2009). Silencing of multiple susceptibility genes (*S*-genes) by RNAi resulted in late blight resistance in potato (Sun et al., 2016). Since RNAi does not always result in a complete knockout, genome-editing could potentially be used to simultaneously knockout genes belonging to the *S*-locus. Recently, an extracellular surface protein called receptor-like protein ELR (elicitin response) from the wild potato species, *S. microdontum*, has been reported to recognize an elicitin that is highly conserved in *Phytophthora* species offering a broad spectrum durable resistance to this pathogen (Du et al., 2015). Introducing both extracellular and intracellular receptors in potato cultivars by genome-editing can aid in attaining durable broad-spectrum resistance for late blight.

Tuber quality traits, such as reduced SGAs or potatoes with reduced bruising, are some of the traits that could be improved using genome-editing. Previously, RNAi silencing of *Polyphenol oxidase* (*PPO*) was shown to reduce the browning in tubers due to mechanical damage (Bachem et al., 1994; Coetzer et al., 2001; Arican and Gozukirmizi, 2003). Recently, anti-browning genetically modified apples have been successfully introduced into market developed by RNAi silencing of *PPO* (Waltz, 2015). Anti-browning mushrooms, developed by targeting *PPO* using CRISPR/Cas9, are not regulated by the USDA, suggesting that traits created by knocking out genes may have an accelerated path to market (Waltz, 2016, 2018).

Reduction of SGA levels in the tuber is another important breeding objective in potato previously achieved by targeting different genes in SGA biosynthetic pathway (Sawai et al., 2014; Cárdenas et al., 2016; Umemoto et al., 2016; McCue et al., 2018). As per industry standards, total glycoalkaloid content must be less than 20 mg/100 g tuber fresh weight to be released for commercial tuber production. However, SGAs also have positive impact as defensive allelochemicals deterring insect herbivores (Sinden et al., 1980; Sanford et al., 1990, 1997). Therefore, reduction of SGAs in aboveground tissues may deteriorate pathogen resistance (Ginzberg et al., 2009). Studies have shown differential levels of SGA accumulation among plant organs and developmental stages (Valkonen et al., 1996; Eltayeb et al., 1997; Friedman and McDonald, 1997). Although α-chaconine and α-solanine, which constitute >90% of SGAs, are the predominant SGAs found in cultivated potato (Moehs et al., 1997; McCue et al., 2005, 2006, 2007), other novel SGAs are found in various *Solanum* species (Shakya and Navarre, 2008; Itkin et al., 2013; Cárdenas et al., 2016). For example, in *S. chacoense*, leptines and leptinines accumulate only in aerial plant organs and are correlated with plant resistance to Colarado potato beetle (Sinden et al., 1980, 1986; Sanford et al., 1990; Mweetwa et al., 2012). Such qualitative differences in SGAs in terms of organ specificity and composition provide opportunities to select specific targets for potato improvement via genome engineering. For example, silencing exclusively tuber expressed members of the SGA biosynthetic pathway or editing specific gene targets expressed in aerial organs can be achieved. The ultimate goal would be to develop new potato cultivars with low SGA levels in tubers while still maintaining high levels in above ground tissues for crop protection.

Breeders are currently working toward re-inventing potato as a diploid crop in order to accelerate progress toward understanding the genetics of complex traits such as yield, quality and drought resistance (Jansky et al., 2016). Genome-editing combined with inbred diploid line development would be a monumental shift in the potential for genetic improvement and opens up possibilities for creating a better potato breeding pipeline. Moving to diploid potatoes enables us to develop hybrids based on selected inbred lines by which we can improve various agronomic traits such as disease resistance and remove compatibility barriers. Genome-editing was successfully applied in diploid potato to overcome gametophytic SI by knocking-out the *Stylar ribonuclease* gene (*S-RNase*) (Ye et al., 2018). Self-compatibility allows fixing of gene edits and segregating out any insertions of foreign DNA from the process of transformation by selection in the progeny. Genome-editing will best be applied to potato improvement using diploid F1 hybrids. There is a need for a set of germplasm that is diploid, inbred and self-compatible forming tubers with commercial shape and appearance and high regeneration capability in plant transformation. Although, some existing diploid lines have some of the characteristics, more work is needed to produce germplasm that meets these requirements.

## Author Contributions

SN and CB conceived the idea, SN wrote most of the manuscript. DV and SN wrote the regulatory aspects. CB, DD, CS, and DV contributed to part of writing and overall improvement of the manuscript. All authors read and approved the manuscript.

## Conflict of Interest Statement

The authors declare that the research was conducted in the absence of any commercial or financial relationships that could be construed as a potential conflict of interest.

## Footnotes

1 http://www.innatepotatoes.com/newsroom/press-releases

2 https://www.aphis.usda.gov/aphis/ourfocus/biotechnology/am-i-regulated

3 https://www.usda.gov/media/press-releases/2018/03/28/secretary-perdue-issues-usda-statement-plant-breeding-innovation

4 https://www.the-scientist.com/news-opinion/japanese-authorities-recommend-not-regulating-gene-editing-64675

5 http://curia.europa.eu/juris/celex.jsf?celex=62016CJ0528&lang1=en&type=TXT&ancre=
